# Extended Kalman Filter (EKF) Design for Vehicle Position Tracking Using Reliability Function of Radar and Lidar

**DOI:** 10.3390/s20154126

**Published:** 2020-07-24

**Authors:** Taeklim Kim, Tae-Hyoung Park

**Affiliations:** 1 Department of Control and Robot Engineering, Chungbuk National University, Cheongju 28644, Korea; taeglem@cbnu.ac.kr; 2 School of Electronics Engineering, Chungbuk National University, Cheongju 28644, Korea

**Keywords:** Kalman filter, sensor fusion, LiDAR, radar

## Abstract

Detection and distance measurement using sensors is not always accurate. Sensor fusion makes up for this shortcoming by reducing inaccuracies. This study, therefore, proposes an extended Kalman filter (EKF) that reflects the distance characteristics of lidar and radar sensors. The sensor characteristics of the lidar and radar over distance were analyzed, and a reliability function was designed to extend the Kalman filter to reflect distance characteristics. The accuracy of position estimation was improved by identifying the sensor errors according to distance. Experiments were conducted using real vehicles, and a comparative experiment was done combining sensor fusion using a fuzzy, adaptive measure noise and Kalman filter. Experimental results showed that the study’s method produced accurate distance estimations.

## 1. Introduction

Vehicle position tracking studies are crucial for accurately estimating distances. Position tracking is used in autonomous vehicle research when solving situations using detection alone is difficult. These types of studies use lidar sensors and radar cameras for detection and recognition. Cameras are useful for object recognition, but there are times when measuring distances robustly is difficult; recent sensor fusion studies attempting robust distance measurements have been undertaken [1,2]. Methods utilizing cameras detect a vehicle from an image, and the distance is estimated by defining a proportional expression between the image coordinate system and the actual distance [3,4,5]. A recent study made use of deep learning to estimate positions; however, this method produced errors when a vehicle passed by on a slope [6].

Camera sensors have difficulties recognizing objects and estimating positions at night, and sensor fusion compensates for these problems by reducing measurement distance errors to improve detection [7,8,9]. Previous studies on vehicle recognition and distance measurement have used a combination of cameras and radar. However, this study used a camera and a radar to track the position of the vehicle. Combining a camera and radar reduces errors compared to using only a single sensor. In sensor fusion research, numerous studies have been conducted on sensor fusion using lidar and cameras dependent on radar sensors for distance measurement [10,11,12,13,14,15,16,17]. Radar sensors are accurate, but errors are evident when used at a close distance. A lidar sensor is used in tandem to minimize errors. In this study, vehicle detection was carried out using lidar and a camera, while radar and lidar sensors were used for distance measurement.

Previous sensor fusion studies using lidar and radar do not reflect the relationship between sensor characteristics and distance [18,19]. Sensor fusion research utilizes methods like decision trees, fuzzy logic, deep learning, and Kalman filters [20,21,22,23]. While the decision tree method cannot represent several situations, fuzzy logic can express various situations with minor difficulties defining membership functions. The deep-learning method shows excellent performance but requires a large amount of computing power. Instead, a Kalman filter was used to track the distance of the target vehicle by combining lidar and radar data.

In this study, a reliability function was designed to reflect the distance characteristics using lidar and radar by analyzing the errors between the sensors according to distance. An extended Kalman filter (EKF) was designed, which confirmed that using the reliability function improved the accuracy of distance estimation.

## 2. Problem Definition

### 2.1. Lidar and Radar Sensor Characteristics According to Target Vehicle Distance

**Figure 1:**
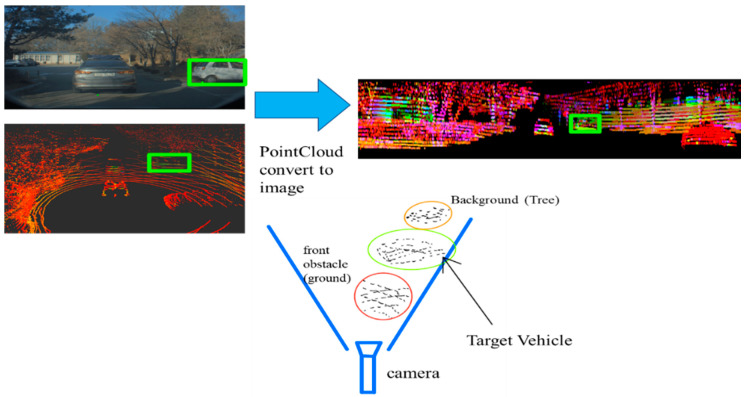
Problems with lidar detection using frustums [29]. Left: Object detected using RGB camera. Right: Detection results in frustum images. Bottom: Obstacles to be removed from frustum image: background (tree) and front obstacle (ground) in the picture.

A lidar sensor displays the reflected object as dots when it hits the object with a laser, and the points measured by the laser produce an unstructured point cloud. Detection through deep learning includes Voxel, MV3D, and Vote3Deep [24,25,26,27]. These deep-learning networks display sufficient performance when using a high-channel lidar. A detection algorithm and an image of the frustum was created because this study used a low-channel lidar to detect the target vehicle [28]. Detection using frustum images presents highlights the problems caused by foreground and background obstructions (Figure 1).

Obstructions are included in the object detection in frustum images. However, a study using an unsupervised learning method, clustering, provided a solution to these problems [29]. We used the following methods to select as many obstacles and object candidates as possible. After changing the point cloud to a top-view image, the threshold removed numerous tree and ground points. Clustering using Euclidean distance constructed the point cloud from candidate vehicles, and conversion to frustum images utilized the candidate’s point cloud. If there is an obstacle in front of the vehicle, boxes of a certain size can be removed, but if a box of the vehicle candidate size is present, these boxes cannot be removed properly. A deep-learning network could solve this problem, but the computing power of the present study’s equipment was insufficient.

**Figure 2:**
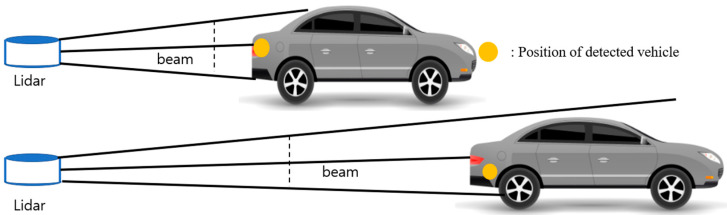
Estimated position of the forward vehicle with a lidar sensor.

Even if the background and obstacles are completely removed, accurately measuring the distance from the detected region of interest (ROI) must be considered. This distance was measured by removing the obstacle from the detected vehicle area and averaging the values at the ROI’s center. However, as the target vehicle moved further away, the measured value produced an error. The lidar data are dependent on various factors, such as the measurement distance and the slope angle of the scanned object [30,31]. A change in the detected vehicle’s lidar beam was considered when the distance changed (Figure 2).

If the target vehicle is close to the sensor or at a high-channel lidar, a relatively accurate value can be obtained by averaging and measuring the detected ROI. However, vehicles far from low-channel lidars have fewer beams reflected, and measuring the distance with a lidar beam in a small area was necessary. This distance was measured by averaging the lidar data measured with the point cloud of the detected ROI. Figure 2 demonstrates that the greater the distance between the target element and the scanner, the greater the mismeasured elements’ effect on the readings, as shown by an error. The lidar can measure closer objects accurately, but the measurement of a distant object is inaccurate.

**Figure 3:**
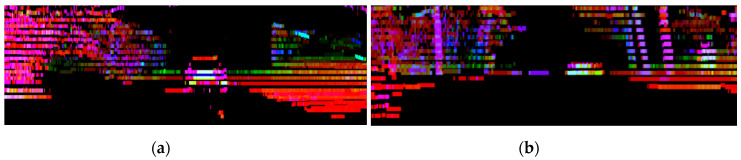
Changes in the frustum image according to the distance of the target vehicle: (**a**) close and (**b**) far.

Figure 3 shows the change in the frustum image’s target vehicle based on distance. Target vehicles at close range can be measured relatively accurately if the center area is averaged after detection. However, if the vehicle is further away, the area will be reduced, and the ROI method will produce inaccurate readings. In particular, a low-channel lidar’s beam decreases rapidly as the distance increases until it cannot be detected. If the vehicle is detected further away, it will appear as a point-cloud line in the frustum image. The point cloud was fused with a radar sensor to reduce the likelihood of errors occurring at long distances.

A radar sensor is not dependent on the time of day and can measure distances more robustly than cameras. Thus, the distance was measured using a camera sensor combined with radar, which is affected by the frequency used or the target object’s speed [32,33]. A radar sensor was installed on the front bumper of the ego vehicle to measure the vehicle’s distance. Although it was measured with an error of 0.2 m, the measurement was inaccurate at a close distance.

**Figure 4:**
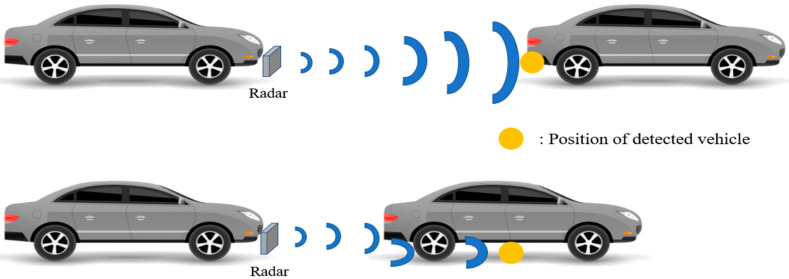
Problem in measurement values with the radar sensor.

The radar confirmed that the lower part of the vehicle was measured from a close distance (Figure 4). Some cases measured radio waves by diffraction from the bottom of the vehicle without returning measurements from the trunk. Distance-specific data were collected to analyze errors and account for the uncertainties of each sensor.

### 2.2. Data Uncertainty Analysis

**Figure 5:**
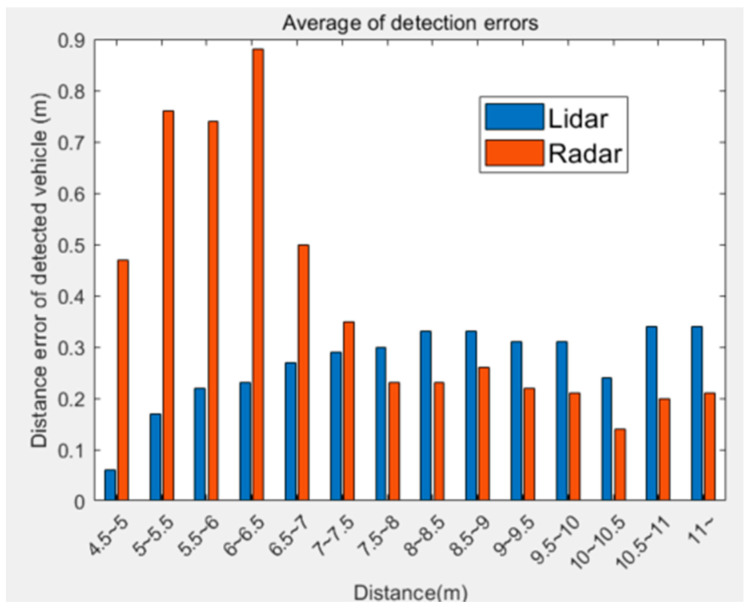
Measurement error lidar sensor and radar sensor according to distance.

The measurement errors of the lidar and radar sensors were calculated and summarized according to distance (Figure 5). Distance errors are based on the sensors’ inputs, and ground truth (GT) manually selects the data measured by lidar. After converting the frustum image into lidar, there was a large error in measuring the distance by averaging the point cloud of the detected vehicle’s ROI. However, the raw point cloud was chosen as GT because it had an error of less than 5 cm. That is, the data error of the unprocessed lidar was less than 5 cm, and the value measured by the lidar was determined as GT. The error was also calculated using Euclidean distance.

Figure 5 summarizes the errors caused by the distance between the lidar and radar sensors, which reflects the characteristics of the sensor, the detector, and other various factors. Upon comparing the distance error, the average lidar error was low at closer distances, and the average of the radar data was low at greater distances. In summary, the radar produced more accurate long-distance measurements, while the lidar more was accurate at close range. An extended Kalman filter with a reliability function was designed to reflect the distance characteristics.

## 3. Vehicle Position Tracking

**Figure 6:**
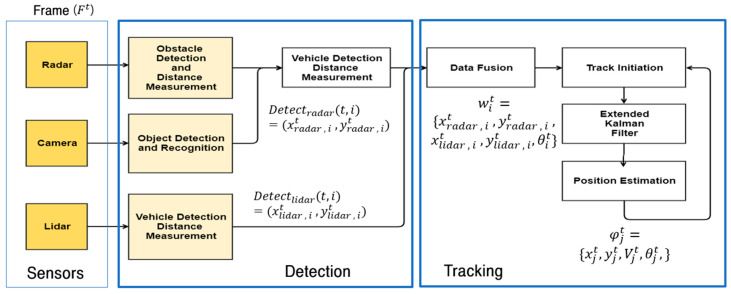
System structure.

The system structure was divided into detection and tracking processes (Figure 6). In the detection process, a camera and a radar sensor were integrated for vehicle recognition and distance measurement purposes, and camera detection was performed using YOLO v3 [34].

The result of the integration of camera and radar sensors is expressed using the formula $Detect_{radar}\left(t,i\right)=\left(x_{radar,\;i}^{t}\;y_{radar,i}^{t}\;\right)$ for I ∈{1…n}, where n is the number of detected objects obtained at time *t*. Lidar detection was performed using convolutional neural networks (CNNs) based on polar view [28]. The detection result of lidar is expressed as follows, using $Detect_{lidar}\left(t,i\right)=\left(x_{lidar\;,i}^{t},y_{lidar\;,i}^{t}\;\right)$ for I ∈{1…n}, where n is the number of detected objects obtained at time *t*. $w_{i}^{t}=\left\{x_{lidar,\;i}^{t},y_{lidar,i}^{t},x_{radar,i}^{t},y_{radar,i}^{t},\theta_{i}^{t}\right\}$ is the detection result. Input $w_{i}^{t}\left(t,i\right)$ was used to predict the next state, and θ is the heading of the vehicle.

Recent studies have shown the possibility of obtaining a vehicle’s heading using a 3D bounding box [35,36]. However, the heading often did not appear when applied correctly to a low-channel lidar. The heading of the vehicle was calculated using the direction accumulated over five frames, the amount of change in each direction was obtained, and θ was calculated using Equation (1) [37].

(1) $$\begin{array}{c} \Delta x_{lidar}=\frac{\sum_{a=2}^{5}\left[\left(a-1\right)\left(x_{lidar\;}^{t-5+a}-x_{lidar}^{t-5+a}\right)\right]}{\sum_{a=2}^{5}\left(a-1\right)},\;\Delta y_{lidar}=\frac{\sum_{a=2}^{5}\left[\left(a-1\right)\left(x_{lidar\;}^{t-5+a}-x_{lidar}^{t-5+a}\right)\right]}{\sum_{a=2}^{5}\left(a-1\right)},\\ \Delta x_{radar}=\frac{\sum_{a=2}^{5}\left[\left(a-1\right)\left(x_{lidar\;}^{t-5+a}-x_{lidar}^{t-5+a}\right)\right]}{\sum_{a=2}^{5}\left(a-1\right)},\Delta y_{radar}=\frac{\sum_{a=2}^{5}\left[\left(a-1\right)\left(x_{lidar\;}^{t-5+a}-x_{lidar}^{t-5+a}\right)\right]}{\sum_{a=2}^{5}\left(a-1\right)},\\ \Delta x_{total}=\frac{\Delta x_{lidar}+\Delta x_{radar}}{2},\;\Delta y_{total}=\frac{\Delta y_{lidar}+\Delta y_{radar}}{2},\\ \theta_{i}^{t}=\mathrm{ATAN}2\left(\Delta y_{total},\Delta x_{total}\right), \end{array}$$

The extended Kalman filter was used to estimate the position and speed of the target vehicle.

### 3.1. Extended Kalman Filter Design

The state variable of the Kalman filter is the jth tracking vehicle result at time t (Equation (2)).

(2) $$\varphi_{j}^{t}=\left\{x_{j}^{t},y_{j}^{t},V_{j}^{t},\theta_{j}^{t}\right\},$$

where x is the front distance from the ego vehicle to the target vehicle, y is the lateral distance from the ego vehicle to the target vehicle, V is the velocity, and θ is the vehicle‘s heading. The Kalman filter‘s measurement vector is the position and direction of the target vehicle. The position and direction are measured through each detector, which determines the measurement vector and gives the position of the ith detection vehicle at time t (Equation (3)).

(3) $$w_{i}^{t}=\left\{x_{lidar,\;i}^{t},y_{lidar,i}^{t},x_{radar,i}^{t},y_{radar,i}^{t},\theta_{i}^{t}\right\},$$

The process model of the state vector is expressed as Equation (4).

(4) $$a_{j}^{t-1}=\left[\begin{array}{c} x_{j}^{t-1}+\Delta tV_{j}^{t-1}\cos\left(\theta_{j}^{t-1}\right)\\ x_{j}^{t-1}+\Delta tV_{j}^{t-1}\sin\left(\theta_{j}^{t-1}\right)\\ \begin{array}{c} V_{j}^{t-1}\\ \theta_{j}^{t-1} \end{array} \end{array}\right],$$

Equation (4) is nonlinear, and was linearized by obtaining the Jacobian matrix in Equation (5).

(5) $$A_{j}^{t-1}=\left[\begin{array}{cccc} 1 & 0 & \Delta tcos\left(\theta_{j}^{t-1}\right) & 0\\ 0 & 1 & \Delta tsin\left(\theta_{j}^{t-1}\right) & 0\\ 0 & 0 & 1 & 0\\ 0 & 0 & 0 & 1 \end{array}\right],$$

Next, $H_{j}$ was defined as a relational expression between the measurement and state vectors. The updated measurement vector was used to build a model that predicted the next state based on the previous state, and the measurement vectors coming from each sensor had different noises depending on the distance. As such, Equation (6) was added to the reliability function.

(6) $$w_{i}^{t}={\left[\begin{array}{c} x_{lidar\;,i}^{t}\\ \begin{array}{c} y_{lidar\;,i}^{t}\\ x_{radar\;,i}^{t}\\ y_{radar\;,i}^{t} \end{array}\\ \theta_{lidar\;,i}^{t} \end{array}\right]}^{T}=[\begin{array}{c} x_{j}^{t-1}+\Delta \text{t}V_{j}^{t-1}\cos\left(\theta_{j}^{t-1}\right)+sig_{\alpha_{1}\beta_{1}}\left(x\right)\\ \begin{array}{c} y_{j}^{t-1}+\Delta \text{t}V_{j}^{t-1}\sin\left(\theta_{j}^{t-1}\right)+sig_{\alpha_{1}\beta_{1}}\left(y\right)\\ x_{j}^{t-1}+\Delta \text{t}V_{j}^{t-1}\cos\left(\theta_{j}^{t-1}\right)+sig_{-\alpha_{2}\beta_{2}}\left(x\right)\\ y_{j}^{t-1}+\Delta \text{t}V_{j}^{t-1}\sin\left(\theta_{j}^{t-1}\right)+sig_{-\alpha_{2}\beta_{2}}\left(y\right) \end{array}\\ \theta_{j}^{t-1} \end{array}]$$

For the lidar sensor, the closer the vehicle was to the front, the more reliable the measured value became, while the opposite was true for the radar sensor. In Equation (6), sig(x) illustrates the reliability function that reflected measurement errors over a distance. The addition of the reliability function made the measurement transformation matrix nonlinear. An extended Kalman filter resolved the equation’s nonlinearity; the next matrix equation was linearized using the Jacobian matrix in Equation (7). The Kalman gain was updated by comparing the predicted value with the measured value using $H_{j}^{t}.$

(7) $$H_{j}^{t}=\frac{\partial h\left(w_{i}^{t}\right)}{\partial w_{i}^{t}}=[\begin{array}{cccc} 1-\left(\alpha_{2}\beta_{2}\right)*sig_{-\alpha_{2}\beta_{2}}^{'}\left(x_{radar,i}^{t}\right) & 0 & \Delta tcos\left(\theta_{j}^{t-1}\right) & 0\\ 0 & 1+\left(\alpha_{1}\beta_{1}\right)*sig_{\alpha_{1}\beta_{1}}^{'}\left(y_{lidar,i}^{t}\right) & \Delta tsin\left(\theta_{j}^{t-1}\right) & 0\\ 1-\left(\alpha_{2}\beta_{2}\right)*sig_{-\alpha_{2}\beta_{2}}^{'}\left(x_{radar,i}^{t}\right) & 0 & \Delta tcos\left(\theta_{j}^{t-1}\right) & 0\\ 0 & 1-\left(\alpha_{2}\beta_{2}\right)*sig_{-\alpha_{2}\beta_{2}}^{'}\left(y_{radar,i}^{t}\right) & \Delta tsin\left(\theta_{j}^{t-1}\right) & 0\\ 0 & 0 & 0 & 1 \end{array}]$$

### 3.2. Reliability Function

**Figure 7:**
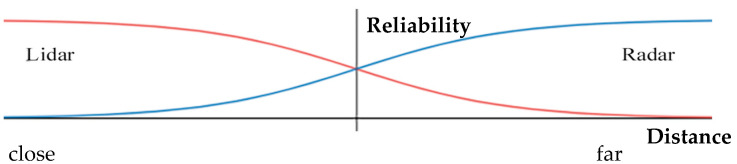
Reliability function; the x-axis is the distance of the opponent vehicle from the ego vehicle. The y-axis represents the reliability of the value measured by the sensor. When comparing the two sensors, the lidar was accurate at close range, and the radar was accurate at long distances.

The position estimation of the vehicle using the Kalman filter predicted the next state using the value measured by the sensor. Numerous factors caused differences between the predicted position and the actual position, such as erroneous measurements. As explained earlier, this study focused on minimizing distance errors by designing a reliability function to reflect the distance characteristics of the sensors in the Kalman filter. In the longitudinal direction, the reliability of the lidar and radar sensors’ values was measured based on distance (Figure 7).

**Figure 8:**
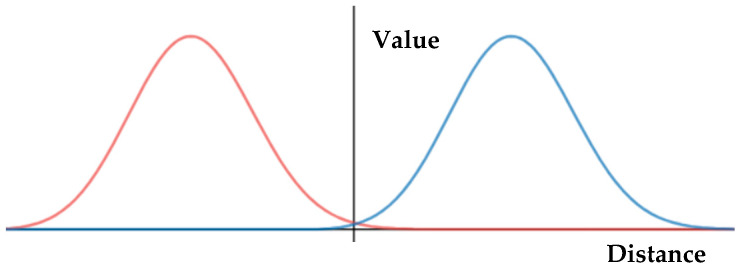
Differentiated reliability function; the x-axis is the distance of the opponent vehicle from the ego vehicle. The y-axis represents the reliability of the value.

The sigmoid function was used to implement the reliability function with Equation (6) by differentiating the matrix, $H_{j}$, which represented the relationship between the state vector and the measurement vector. Figure 8 shows the differential of the sigmoid function.

The differential sigmoid function had the same distribution as the Gaussian function, which reflected the measurement vector’s errors. Meanwhile, the reliability function was expressed as Equation (8). In general, a Kalman filter analyzes the errors of each sensor and reflects measurement noise using a Gaussian function. Measurement noise should analyze and identify as many errors as possible, but this is not easily performed. A direct substitution into the measurement transformation matrix aimed to reflect this error.

(8) $$\begin{array}{c} sig_{\alpha_{1}\beta_{1}}\left(x\right)=\frac{\beta_{1}}{1+e^{\alpha_{1}\left(x-X_{lidar,\;\;reli}\right)}},\\ sig_{-\alpha_{2}\beta_{2}}\left(x\right)=\frac{\beta_{2}}{1+e^{-\alpha_{2}\left(x-X_{radar,\;reli}\right)}},\\ sig_{\alpha_{1}\beta_{1}}\left(y\right)=\frac{\beta_{1}}{1+e^{\alpha_{1}\left(y-Y_{lidar,\;\;reli}\right)}},\\ sig_{-\alpha_{2}\beta_{2}}\left(y\right)=\frac{\beta_{2}}{1+e^{-\alpha_{2}\left(y-Y_{radar,\;reli}\right)}}, \end{array}$$

As shown in Figure 5, the sensors’ measurement errors did not exactly switch between one another. The distance characteristics of each sensor did not cross the middle value. Therefore, the sections where the sensor’s error and reliability function’s value were at the maxima had to match, which changed the reliability function of each sensor with parameters $X_{lidar,reli}$ and $X_{radar,reli}$. The uncertainty of the measured value increased from half the maximum detection distance. Each variable corresponded with a point where the reliability of a sensor was halved. This variable was expressed as the probability of a more reliable sensor among the two; $\alpha_{1}$, $\alpha_{2}$, $\beta_{1}$, $\beta_{2}$ were determined through experiments.

**Figure 9:**
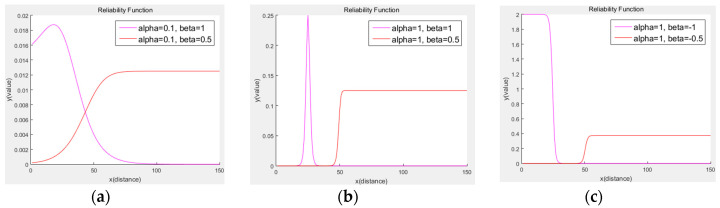
Differentiation result of the reliability function according to parameter change: when $X_{lidar,reli}=25$, $X_{radar,reli}=50,$ (**a**) α = 0.1, β = 1 and α = 0.1, β = 0.5; (**b**) α = 1, β = 1 and α = 1, β = 0.5; (**c**) α = 1, β = −1 and α = 1, β = −0.5.

Figure 9 shows the differential result of the reliability function for each parameter change. The x-axis is the distance value measured by the sensor, and the y-axis is the output value of each parameter when input is added to the reliability function. The uncertainty of each sensor, according to the measured value, is reflected in the Kalman filter. $H_{j}^{t-1}$ changes adaptively and updates the Kalman filter using the reliability function.

### 3.3. Kalman Filter Update

The state vector $\varphi_{j}^{t}$ was estimated using the state transition matrix $A_{j}^{t-1}$ and expressed as Equation (9).

(9) $$\varphi_{\bar{j}}=A_{j}^{t-1}\varphi_{j}^{t-1}$$

Next, the error covariance $P_{j}^{t}$ was obtained.

(10) $$P_{j}^{t}=A_{j}^{t-1}P_{j}^{t-1}A_{j}^{t-1^{T}}+Q,$$

In Equation (11), Q is defined as system noise. The Kalman gain was obtained using the updated error covariance $P_{j}^{t}$ and the measurement transition matrix $H_{j}^{t-1}$.

(11) $$K_{j}^{t}=P_{j}^{t}H_{j}^{t-1^{T}}{\left[H_{j}^{t-1}P_{j}^{t}H_{j}^{t-1^{T}}+R_{j}\right]}^{-1},$$

For Equation (12), $R_{j}$ is the measurement noise. The Kalman gain, the measured sensor value, and the predicted value were used to obtain $\varphi_{j}^{t}$, thus estimating the next value of the state vector.

(12) $$\varphi_{j}^{t}=\varphi_{j}^{t-1}+K_{j}^{t}\left[w_{j}^{t}-H_{j}^{t-1}\varphi_{j}^{t}\right],$$

In Equation (13), updates of the error covariance were obtained using the updated Kalman gain.

(13) $$P_{j}^{t}=\left[I-K_{j}^{t}H_{j}^{t}\right]P_{j}^{t-1},$$

### 3.4. Tracking Management

**Figure 10:**
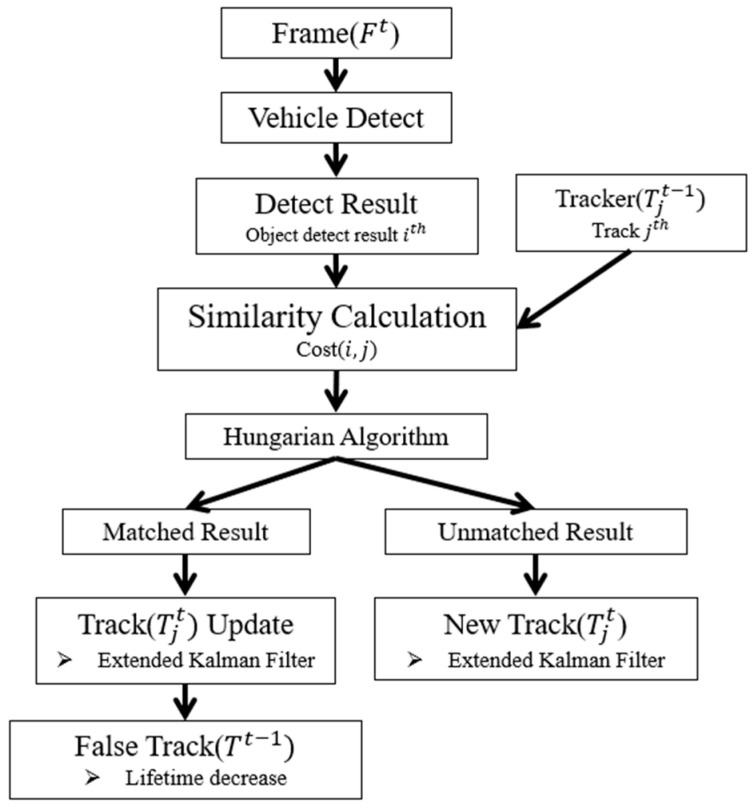
Tracking management.

Tracking management (Figure 10) was designed for experiments with multiple vehicles. The experimental section describes the use of real vehicles during the experiments, while multiple vehicles were used in a simulation. In the latter case, the detection and tracking results should match. The following is an explanation of the detection and tracking results.

The detection results of the lidar and radar obtained at the time *t* were used as the inputs. When the detected vehicle was called $Detect_{i}\left(x,y\right)$, the contents of the detected vehicle were expressed as Equation (14). $Detect_{i}\left(x,y\right)$ includes the x and y positions and θ. Even if only one sensor obtained a detection result, the vehicle tracker was updated. θ was measured in the manner previously described. If only the lidar or radar data were measured, θ was obtained using only one sensor value.

(14) $$Detect_{i}\left(x,y,\theta \right)=\{\begin{array}{c} x_{lidar}^{i},\;y_{lidar,\;i}^{t},\;\left(only\;lidar\right)\\ x_{radar}^{i},\;y_{radar,\;i}^{t},\;\left(only\;radar\right)\\ \theta_{i}^{t}, \end{array}$$

The detection result used as input calculated the similarity to the $T_{j}^{t-1}$ obtained by the tracker Equation (15).

(15) $$T_{j}^{t-1}\left(x,y,V,\theta \right)=\{\begin{array}{c} \varphi_{j}^{t-1}=\left\{x_{j}^{t-1},y_{j}^{t-1},V_{j}^{t-1},\theta_{j}^{t-1}\right\}\\ Lifetime=k,\;k<Maxlife \end{array},$$

The similarity was calculated using two matching pieces of information (Equation (16)), and the similarity calculation used Euclidean distance.

(16) $$cost\left(Detect_{i},T_{j}^{t-1}\right)=\sqrt[{2}]{{\left(Detect_{i}\left(x\right)-T_{j}^{t-1}\left(x\right)\right)}^{2}+{\left(Detect_{i}\left(y\right)-T_{j}^{t-1}\left(y\right)\right)}^{2}}$$

Data association used a Hungarian algorithm, frequently used for allocation problems where cost must be lower than the threshold to allocate [38]. For example, if the threshold is 1 and the cost is 2, it is not assigned. However, if the cost is greater than the threshold, it is considered irrelevant. Thus, all detectors calculated the cost of each existing tracker and matched them accordingly. The unassigned result proceeded as follows. If the detection result was not matched with the existing tracker using the algorithm, a new tracker was created. The matched tracker updated the Kalman filter and increased the tracker’s lifetime; unassigned trackers had reduced lifetimes.

## 4. Experiment

**Figure 11:**
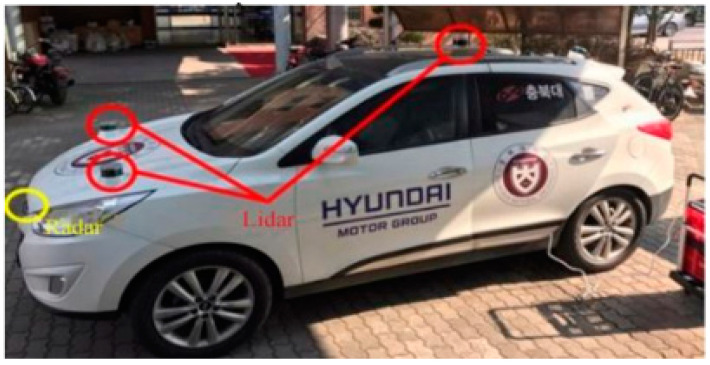
Test vehicle. It was equipped with three lidars and one radar.

Figure 11 shows the vehicle used in the experiment, which was equipped with three Velodyne VLP-16 lidar sensors. Attached to the front of the vehicle was a Delphi ESR 2.5 radar sensor, and the coordinate system was calibrated based on the left lidar sensor. The lidar data used the detected data for the vehicles after converting the point cloud to the spherical coordinate system. Experiments were then performed using the fuzzy method and the proposed method to reflect the sensor’s distance characteristics accurately. For system noise and measurement noise, a Kalman filter accurately estimated the process’s variables [39].

Table 1 shows the noise settings of each filter used. When the measured value was affected by the same value, the experiment was conducted by setting the same parameters as follows to confirm the switching effect. However, in the case of adaptive noise, the experiment was conducted by multiplying the coefficients and changing them according to the switching situation.

### 4.1. Fuzzy Rule

The fuzzy algorithm sets fuzzy rules and membership functions that represent various situations. This study used sensor fusion while considering distance characteristics. Fuzzy rules were based on this idea and are shown in Table 2.

**Figure 12:**
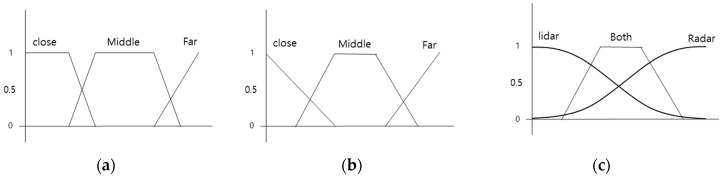
Membership function: (**a**) lidar membership function; (**b**) radar membership function; (**c**) output membership function.

When the target vehicle was close, the lidar measurement was more reliable than radar; at greater distances, radar was more reliable than lidar. This relationship served as the basis of the fuzzy rules. The output $f_{o}$. $x_{m}$ was the median of the maximum distances both sensors could detect. If it was below $x_{m}$, the output value was determined by the weight of the lidar. However, if detected data from lidar were more than or equal to $x_{m}$, it was used as the radar’s weight Equation (17). Figure 12 is a membership function of the fuzzy algorithm. The fuzzy membership function was used as a reference, and the parameters were changed during the experiment [40].

(17) $$\begin{array}{c} f_{o}*x_{lidar,i}+\left(1-f_{o}\right)*x_{radar,i}=x_{f}\;\left(if\;x_{lidar\;,i}<x_{m}\right),\\ f_{o}*y_{lidar\;,i}+\left(1-f_{o}\right)*y_{radar\;,i}=y_{f},\\ \left(1-f_{o}\right)*x_{lidar\;,i}+f_{o}*x_{radar,i}=x_{f}\;\left(if\;x_{lidar\;,i}\geq \;x_{m}\right),\\ \left(1-f_{o}\right)*y_{lidar\;,i}+f_{o}*y_{radar\;,i}=y_{f}, \end{array}$$

### 4.2. In Reality for a Single Vehicle

**Figure 13:**
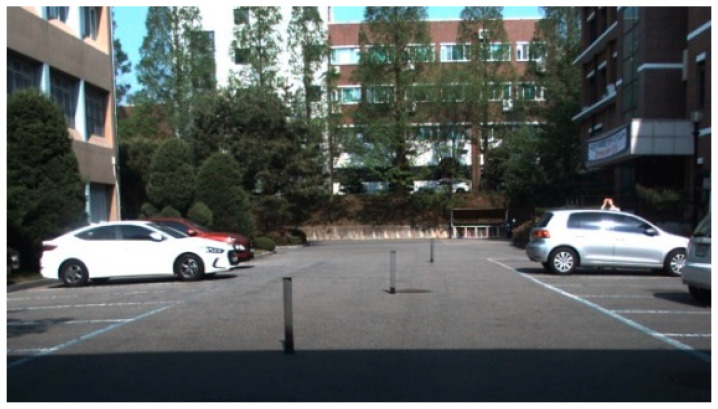
Test vehicle equipped with three lidars, one radar, and a dashboard camera.

Object tracking was performed using an experimental vehicle that compared the sensor fusion of the camera and radar and the fusion of the camera lidar sensor. The distance between the camera and the radar was calibrated by using the coordinates of the pillars in the image to obtain a matrix that converted the value of the image pixel into a radar distance coordinate system [41]. The camera computed the projective transformation matrix between the reference object’s x and y coordinates and the distance measured by the radar. Figure 13 shows the image of calculation of the projection transformation matrix.

**Figure 14:**
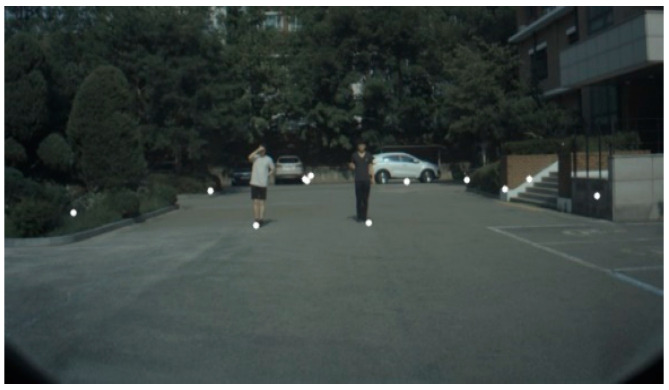
The result of projecting the radar detection result in the image coordinate system.

The measurements used a thin object to maintain the accuracy of the camera and radar’s projection transformation matrix. For these thin objects, the matrix was computed using the image’s x and y ground coordinates, and the radar detection results were projected onto the image coordinate system using the calculated matrix (Figure 14).

The perspective transformation matrix allowed the measurement of distances by comparing the results detected in the image with the radar detection results, which were also obtained using the above method. The result of the radar camera sensor fusion and lidar camera sensor fusion was expressed as Equation (18), and the algorithm used in the experiment was a Kalman filter. Said equation is equal to Equation (18).

$$\varphi_{j}^{t}=\left\{x_{j}^{t},y_{j}^{t},V_{j}^{t},\theta_{j}^{t}\right\}$$

$$w_{i}^{t}=\left\{x_{lidar,\;i}^{t},y_{lidar,i}^{t},\theta_{\;i}^{t}\right\}\;\left(\mathrm{In}\;\mathrm{case}\;\mathrm{of}\;\mathrm{sensor}\;\mathrm{fusion}\;\mathrm{of}\;\mathrm{lidar}\;\mathrm{and}\;\mathrm{camera}\right)$$

(18) $$w_{i}^{t}=\left\{x_{radar,i}^{t},y_{radar,i}^{t},\theta_{\;i}^{t}\right\}\;\left(\mathrm{In}\;\mathrm{case}\;\mathrm{of}\;\mathrm{sensor}\;\mathrm{fusion}\;\mathrm{of}\;\mathrm{radar}\;\mathrm{and}\;\mathrm{camera}\right)$$

$$A_{j}^{t-1}=\left[\begin{array}{cccc} 1 & 0 & \Delta tcos\left(\theta_{j}^{t-1}\right) & -\Delta tV_{x,j}^{t-1}\sin\left(\theta_{j}^{t-1}\right)\\ 0 & 1 & \Delta tsin\left(\theta_{j}^{t-1}\right) & \Delta tV_{x,j}^{t-1}\cos\left(\theta_{j}^{t-1}\right)\\ 0 & 0 & 1 & 0\\ 0 & 0 & 0 & 1 \end{array}\right],$$

$$H_{j}^{t-1}=\frac{\partial h\left(w_{i}^{t}\right)}{\partial w_{i}^{t}}=\left[\begin{array}{ccc} 1+\Delta tV_{j}^{t-1}\cos\left(\theta_{j}^{t-1}\right) & 0 & -\Delta tV_{x,j}^{t-1}\sin\left(\theta_{j}^{t-1}\right)\\ 0 & 1+\Delta tV_{j}^{t-1}\sin\left(\theta_{j}^{t-1}\right) & \Delta tV_{x,j}^{t-1}\cos\left(\theta_{j}^{t-1}\right)\\ 0 & 0 & 1 \end{array}\right],$$

The data used in the study were obtained using the experimental vehicle. A total of 17 data scenarios were generated by combining left and right turns and approaching or moving away from the ego vehicle. GT was determined manually by viewing the lidar data, and the distance accuracy was evaluated by calculating the GT and the root-mean-squared error (RMSE) of the tracking results. Table 3 shows the results of comparing the distances between different sensor fusions. As a comparative experiment, fuzzy and adaptive measurement noises were used, and the results confirmed that the proposed method was more accurate at estimating positions by showing the errors according to the distance of the sensor.

**Figure 15:**
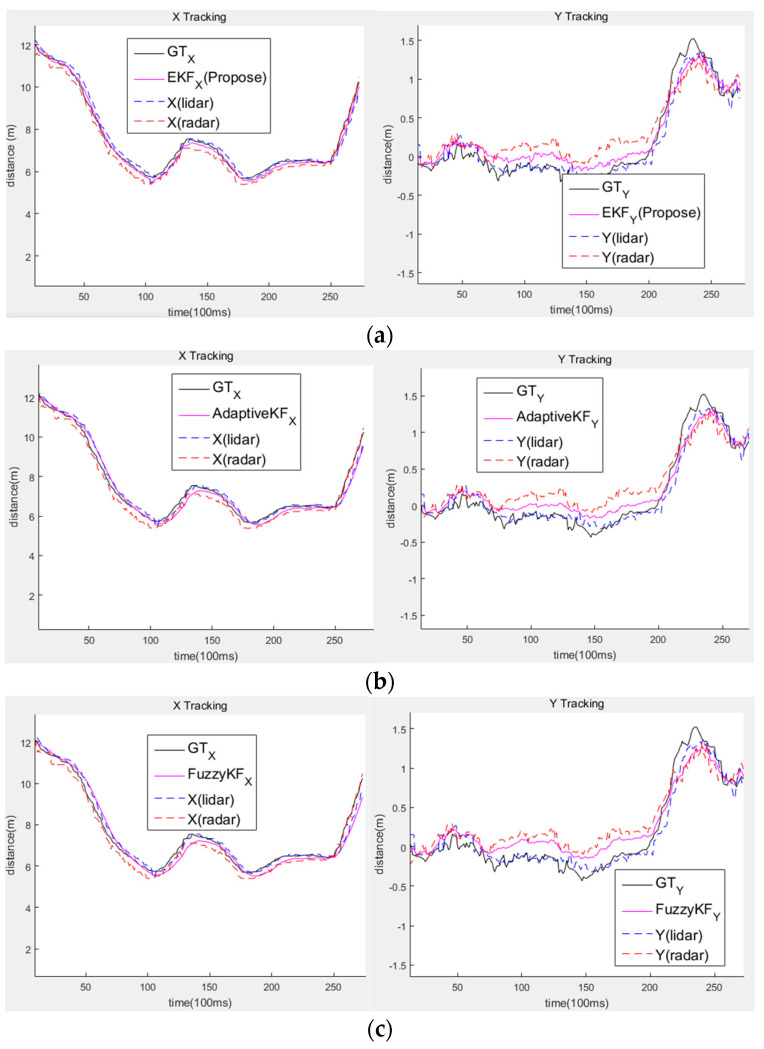
Comparison experiment result of other sensor fusion methods: (**a**) proposed method; (**b**) adaptive measure noise and Kalman filter; (**c**) fuzzy method and Kalman filter.

Figure 15 also shows that the method proposed in this study reflected more accurate distance measurements and position estimation results. The distance characteristics of the sensor made accurately measuring distance characteristics challenging, and changing the measurement noise made estimating accurate distances difficult because the error covariance update was constantly changing. Therefore, the method proposed in this study was suitable for reflecting distance characteristics.

### 4.3. In Simulation for Multiple Vehicles

**Figure 16:**
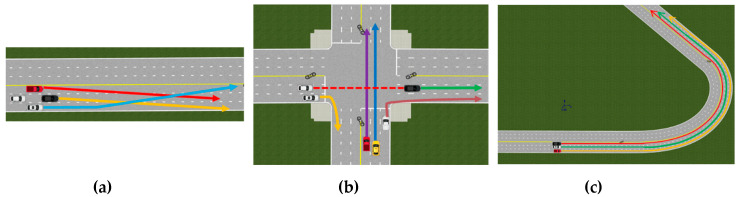
Simulation of three scenarios: (**a**) vehicles changing lanes; (**b**) intersection scenario; (**c**) curve scenario.

As testing multiple vehicles in real-world environments was difficult, experiments were conducted using simulations. The data were generated using a Prescan simulator with multiple vehicles in three different scenarios (Figure 16). Sensor fusion and comparison experiments were carried out using the fuzzy algorithm.

The first scenario showed several vehicles changing lanes; the proposed method was similar to GT (Table 4). The multiple object tracking precision (MOTP) calculated and checked the precision of the estimated tracked position [42]. Using the calculated results, the method proposed in the first scenario reflected accurate position estimations.

The next scenario illustrated a vehicle passing an intersection. In this case, the vehicle’s lateral movement was tracked. Table 5 shows the results in Scenario2. For the y-axis movement, the proposed method was imprecise. However, the proposed method provided an estimate close to the GT along the x-axis.

The final scenario showed the vehicle moving closer to and further away from a curve. As it moved closer, then further away, the data obtained by the sensors were inaccurate. When the vehicle moved away from or close to the ego vehicle, the values measured by the lidar or radar sensors were imprecise. In this scenario, the simulation data were generated, and the experimental results showed that the proposed method was more precise than the sensor fusion using fuzzy results. The results of the experiment are summarized in Table 6. Finally, calculating the MOTP by reflecting each situation frame by frame showed that the proposed method reduced errors by 0.22 m. The results of all scenarios are shown in Table 7.

The above results confirmed that the distance estimation along the x-axis was more accurate. Although the simulation and actual vehicle test results were similar, the y-axis method was correct, but the performance only showed a small improvement.

## 5. Conclusions

In this study, a fused sensor combined the characteristics of lidar and radar sensors according to distance. A point cloud, measured with lidar, provided a top-view image that removed ground and obstacle points before being converted into a frustum image. Meanwhile, the radar was fused with the camera for detection and distance measurement. The lidar obtained accurate measurements when objects were close to it, and the radar sensor was accurate when measuring distant objects. An extended Kalman filter was constructed to reflect the characteristics of each sensor while obtaining measurements. A combination of the two sensors was created using a Kalman filter, which was designed as an extended filter that reflected distance characteristics by adding a reliability function. The experiment utilized an actual vehicle to evaluate the method’s performance. While estimating the distance by identifying the characteristics of the sensor, the system noise of the Kalman filter was compared with a fuzzy method or the proposed method. The study confirmed that the accuracy of distance measurements was improved as a result of the lidar and radar sensor fusion, and the method that reflected distance errors was more accurate in the extended Kalman filter’s composition.

## Figures and Tables

**Table 1:** Noise covariances of the proposed algorithm.

**Q (Process model noise)**	EKF (Proposed) Fuzzy Adaptive	diag (0.5, 0.05, 0.05, 0.05)
**R (Measurement noise)**	EKF (Proposed)	diag (0.05, 0.05, 0.05, 0.05, 0.05)
	Fuzzy	diag (0.05, 0.05, 0.05)
	Adaptive	α*diag (0.05, 0.05, 0.05)

**Table 2:** Fuzzy rules.

Input		Output
Lidar	Radar	Output Weight
Close	Close	Lidar
	Middle	Lidar
	Far	Both
Middle	Close	Lidar
	Middle	Both
	Far	Radar
Far	Close	Both
	Middle	Radar
	Far	Radar

**Table 3:** Comparison of the results of the proposed method with other sensor fusion methods.

Method RMSE (Frame)	Proposed Method		Adaptive Measure Noise		Fuzzy	
	Lidar/Radar/Camera		Lidar/Radar/Camera		Lidar/Radar/Camera	
	x (m)	y (m)	x (m)	y (m)	x (m)	y (m)
Far (681)	0.29	0.30	0.41	0.38	0.28	0.34
Close (547)	0.32	0.22	0.33	0.22	0.32	0.29
Left turn (152)	0.55	0.35	0.54	0.36	0.61	0.33
Right turn (210)	0.87	0.31	0.91	0.31	1.07	0.31
Left curve (210)	0.28	0.20	0.41	0.24	0.46	0.25
Right curve (72)	0.39	0.25	0.47	0.30	0.25	0.22
Total (1882)	0.38	0.27	0.50	0.33	0.40	0.30

**Table 4:** Comparison of the results of the proposed method with fuzzy method in Scenario 1.

Method MOTP (Frame)	Proposed Method		Fuzzy Method	
	Lidar/Radar/Camera		Lidar/Radar/Camera	
	x (m)	y (m)	x (m)	y (m)
Vehicle 1 (112)	1.12	1.04	1.19	1.02
Vehicle 2 (65)	1.08	0.63	1.25	0.68
Vehicle 3 (128)	0.93	0.67	1.04	0.66
Total	1.01	0.65	1.20	0.67

**Table 5:** Comparison of the results of the proposed method with fuzzy method in Scenario 2.

Method MOTP (Frame)	Proposed Method		Fuzzy Method	
	Lidar/Radar/Camera		Lidar/Radar/Camera	
	x (m)	y (m)	x (m)	y (m)
Vehicle 1 (60)	1.07	0.64	1.36	0.67
Vehicle 2 (67)	1.12	0.91	1.17	0.89
Vehicle 3 (40)	1.07	0.53	1.27	0.51
Total	1.09	0.72	1.26	0.72

**Table 6:** Comparison of the results of the proposed method with fuzzy method in Scenario 3.

Method MOTP (Frame)	Proposed Method		Fuzzy Method	
	Lidar/Radar/Camera		Lidar/Radar/Camera	
	x (m)	y (m)	x (m)	y (m)
Vehicle 1 (155)	1.12	0.35	1.42	0.32
Vehicle 2 (137)	0.93	0.33	1.20	0.31
Total	1.03	0.72	1.32	0.72

**Table 7:** Comparison of the results of the proposed method with fuzzy method in all scenarios.

Method MOTP (Frame)	Proposed Method		Fuzzy Method	
	Lidar/Radar/Camera		Lidar/Radar/Camera	
	x (m)	y (m)	x (m)	y (m)
Total	1.04	0.69	1.26	0.70
